# COVID-19 Disease Burden in the Omicron Variant-Dominated Endemic Phase: Insights from the ROUTINE-COV19 Study Using Real-World German Statutory Health Insurance Data

**DOI:** 10.3390/v17030424

**Published:** 2025-03-14

**Authors:** Sabrina Müller, Andrea Schmetz, Julia K. Knaul, Thomas Wilke, Jingyan Yang, Sabine Dornig, Clara Lehmann, Christoph D. Spinner

**Affiliations:** 1 GIPAM GmbH, 23966 Wismar, Germany; 2 BioNTech Europe GmbH, 10179 Berlin, Germany; andrea.schmetz@biontech.de (A.S.); julia.knaul@biontech.de (J.K.K.); 3 Institut für Pharmakoökonomie und Arzneimittellogistik (IPAM), 23966 Wismar, Germany; thomas.wilke@ipam-wismar.de; 4 Pfizer Inc., New York, NY 10017, USA; jingyan.yang@pfizer.com; 5 AOK PLUS, 07743 Jena, Germany; sabine.dornig@plus.aok.de; 6 Department of Internal Medicine, Faculty of Medicine and University Hospital Cologne, University of Cologne, 50937 Cologne, Germany; clara.lehmann@uk-koeln.de; 7 Department of Clinical Medicine, University Medical Center, Technical University of Munich, 81675 Munich, Germany; christoph.spinner@mri.tum.de

**Keywords:** COVID-19 endemic phase, COVID-19 case severity, in-hospital COVID-19 case mortality, healthcare burden, economic impact, statutory health insurance data Germany

## Abstract

The ROUTINE-COV19 study explores the burden of COVID-19 in Germany during the early endemic phase, assessing disease patterns and their impact on the healthcare system from 1 July 2022 to 30 June 2023. Using anonymized statutory health insurance data from over 3 million individuals in Thuringia and Saxony, COVID-19 cases were identified through diagnostic codes, with severe and critical cases defined by hospitalization and intensive care criteria. The study focused on high-risk populations as identified by the German Immunization Technical Advisory Group. During the study period, 414,648 new COVID-19 cases were documented, with peaks in October 2022 and March 2023. Severe cases occurred at a rate of 241.6 per 100,000 persons, with in-hospital mortality exceeding 12%. Critical cases requiring intensive care had an in-hospital mortality rate of 32.2%. COVID-19-related hospitalizations averaged 9.94 days, generating direct costs of EUR 64.9 million, while indirect costs from work absenteeism amounted to EUR 454.3 million, representing 7.5% of all-cause absenteeism costs. Despite entering an endemic phase, COVID-19 continues to pose a substantial burden, particularly among older adults and those with pre-existing cardiovascular conditions.

## 1. Introduction

The coronavirus disease 2019 (COVID-19) pandemic, caused by the SARS-CoV-2 virus, has profoundly impacted public health systems and economies worldwide. Since its emergence in late 2019, successive infection waves within the pandemic have posed ongoing challenges to healthcare providers, policymakers, and researchers [1,2,3]. However, as vaccination campaigns have been rolled out globally and new variants of the virus have emerged, the nature of COVID-19 has transitioned from an acute high-mortality pandemic into a more endemic disease pattern [4].

The COVID-19 pandemic began in late 2019 and by March 2020, the World Health Organization (WHO) declared a global pandemic [5,6]. In Germany, as in many other countries, the early phase of the pandemic was marked by widespread public health measures such as lockdowns, social distancing, and mask mandates to control the spread of the virus [5]. The initial strain of SARS-CoV-2 led to high rates of severe illness and mortality, particularly among older adults and those with underlying health conditions. In late 2020 and early 2021, new dominant SARS-CoV-2 variants emerged as a result of the evolution of SARS-CoV-2. German COVID-19 incidence and prevalence data published by the German national centers for disease control, the Robert Koch Institute (RKI), depict significant and recurring waves [7]. The economic impact of these recurring infection waves and their sequelae have been further quantified, estimating 10 to 15 days of work absenteeism due to COVID-19 in non-hospitalized and hospitalized individuals, respectively [8]. The production loss associated with post- or long COVID in the pandemic timeframe was estimated to be EUR 5.7 billion in Germany [9]. Toward the end of 2021, the Omicron variant (B.1.1.529) was identified and quickly became the dominant strain globally, including in Germany [10]. Omicron variants spread much faster than previous variants but are generally associated with milder symptoms, particularly among vaccinated individuals [11,12,13]. Nevertheless, its high transmissibility caused a substantial number of infections, leading to high caseloads in early 2022; however, the rate of severe cases and deaths was lower than during previous waves [14]. Omicron’s subvariants continued circulating throughout 2022 and 2023 as the virus transitioned from a pandemic to an endemic phase in many regions [15,16]. To date, Omicron subvariants remain the predominant strains in Germany and Europe, steering the course of the endemic evolution of COVID-19.

The ROUTINE-COV19 study was initiated in Germany to provide real-world insights into the evolving burden of COVID-19 in the endemic setting, focusing on changes in disease severity risk stratification and the economic impact of the post-pandemic Omicron variant. By utilizing statutory health insurance (SHI) data covering a broad cross-section of the German population, this study aims to identify patterns of disease burden and assess the impact on healthcare services during the first year of endemicity from July 2022 to June 2023. This analysis characterizes the epidemiological trend associated with SARS-CoV-2 in the post-pandemic period, identifies frequencies of different disease severities, and highlights the ongoing healthcare burden posed by COVID-19 in its endemic phase.

## 2. Materials and Methods

### 2.1. Data Source

This analysis is part of the ongoing ROUTINE-COV19 study, a real-world retrospective cohort analysis designed to assess the clinical and economic impact of COVID-19 in Germany during the endemic phase of the SARS-CoV-2 virus. The study utilized SHI data from the German federal states of Thuringia and Saxony (AOK PLUS), covering more than 3 million individuals.

German claims data provide information on patient demographics (age, gender, date of death) and detailed reimbursement claims on inpatient care and outpatient care, including pharmaceutical treatments and therapeutic devices. Inpatient care data cover information on the date of admission and discharge, procedures (according to the operation and procedure coding, the “Operationen-und Prozedurenschlüssel (OPS)” [17]), the main diagnosis of the hospitalization, and further primary and secondary diagnoses. Outpatient care data comprise information on diagnostic and therapeutic procedures according to the German Uniform Valuation Scheme (EBM [18]), the diagnosis made by an outpatient physician, and the specialty of the treating physician. Inpatient and outpatient diagnoses are coded according to the German Modification of the International Classification of Diseases, Tenth Revision, (ICD-10-GM).

The SHI data were supplemented with physician survey and medical chart data to verify the diagnostic codes used and enhance the accuracy of the claims data analysis.

### 2.2. Study Population

The study population comprised all adults insured by AOK PLUS on 1 July 2022. Individuals who did not have continuous insurance coverage for the 12 months prior to the index date were excluded from the analysis to ensure sufficient data for baseline risk stratification. Individuals were followed until 30 June 2023 so that the study period reflected the first year of endemic COVID-19 in Germany.

The overall population was further categorized into the following specific risk groups:

- Older adults, defined as individuals aged 60 years or older.
- Cardiovascular (CV) risk population, defined as individuals with a CHA2DS2-VASc score ≥ 3 or those diagnosed with atrial fibrillation (ICD-10-GM: I48.0/1/2/9), coronary heart disease (ICD-10-GM: I20, I21-I22, I24, I25), or heart failure (ICD-10-GM: I50), identified by two confirmed outpatient diagnoses in two different quarters or one inpatient diagnosis using the respective ICD-10-GM codes in the 12-month pre-index period.
- Immunocompromised individuals as defined by the German Standing Committee on Vaccination (STIKO, see Supplemental Table S1 for specific conditions and code).
- Individuals suffering from other non-immunocompromising STIKO risk conditions (see Supplemental Table S1).

### 2.3. Outcomes

The primary outcome of interest was the incidence of COVID-19 cases, with a particular focus on severe, critical, and fatal courses of disease. Disease burden is most significant for these individuals, their caregivers, and the healthcare system, and efforts are made to prevent severe disease. A new COVID-19 case was defined by a confirmed outpatient or inpatient diagnosis of ICD-10-GM code U07.1! or U07.2!, with a gap of at least six weeks from the previous U07.1!/U07.2!-related claim (based on the date of the last documented EBM of a different outpatient case number or discharge date for inpatient cases). COVID-19 cases were classified into non-severe, severe, and critical cases as follows:

- Non-severe cases: COVID-19 cases that did not require hospitalization, i.e., individuals with a confirmed outpatient diagnosis of COVID-19 without subsequent hospital admission.
- Severe cases: Hospital admissions with a confirmed COVID-19 diagnosis (ICD-10-GM U07.1!) and at least one of the following conditions:
  ◦ A predefined main diagnosis indicating severe disease (pneumonia, chronic disease of the lower respiratory tract, respiratory infections, heart failure, chronic heart disease, acute pericarditis/myocarditis, or atrial fibrillation; see Supplemental Table S1 for respective ICD-10-GM codes).
  ◦ A requirement for mechanical ventilation, regardless of the main diagnosis (OPS codes 8-711, 8-712, 8-713, 8-714).
- Critical cases: A subset of severe cases that required intensive care (OPS codes 8-980, 8-97a, 8-97b, 8-98d, 8-98f, 8-712.0, 8-721.1, 8-721.2, 8-721.3).

A fatal clinical course was defined as in-hospital death in patients classified as having severe COVID-19 cases.

Patient characteristics were based on claims in the 12-month pre-index period before the index date (1 July 2022). Data included demographics, comorbidity profiles, and pre-existing medical conditions, which were analyzed to compare general COVID-19 cases with severe and critical cases. Demographic variables included age, gender, and employment status. Comorbidity profiles were assessed using both the Charlson Comorbidity Index (CCI [19]; refer to Supplemental Table S2) and the Elixhauser Comorbidity Index (Supplemental Table S3, [20]). Additionally, the CHA_2_DS_2_-VASc score (Supplemental Table S4, [21]) and the presence of specific CV diagnoses (atrial fibrillation, heart failure, coronary heart disease) were used to assess CV risk. The presence of immunocompromised status, other STIKO high-risk conditions, and depression and anxiety disorders were also described and compared between groups.

Secondary outcomes included the impact of COVID-19 on overall healthcare utilization, such as the number of hospitalization days, outpatient visits related to a COVID-19 diagnosis (approximated by the number of dates invoiced for EBM codes), rehabilitation days, and the economic burden associated with healthcare resource utilization in terms of direct costs to the SHI and indirect costs related to work absences. In Germany, the reimbursement of outpatient services is regulated by the EBM system, which assigns weighted points rather than direct monetary values to services. To estimate outpatient care cost, the weighted points were multiplied by a uniform orientation value set by the National Association of Statutory Health Insurance Physicians. Inpatient costs, covering all services and administered drugs during hospital stays, were calculated according to the diagnosis-related group (DRG) system. Indirect costs related to sick leave for individuals aged 18 to 66 years were estimated by multiplying the number of days of absence from work due to COVID-19, as recorded in the system (ICD-10-GM codes: U08.9, U09.9!, U10.9, U07.1!, or U07.2!), by the average daily productivity loss per worker in Germany, as reported by the Federal Institute for Occupational Safety and Health (BAuA [22]).

### 2.4. Statistical Analysis

The frequency of observed COVID-19 cases was expressed as a rate per 100,000 persons and descriptively reported for the overall population and the different pre-defined risk populations. The crude rates of any, severe, critical, and fatal COVID-19 cases as well as age- and gender-standardized numbers were calculated for the overall population. Age- and gender-standardized estimates were based on the German SHI population (reference population) using the KM6 statistic reported by the Federal Ministry of Health.

Descriptive statistics were used to report the characteristics of non-severe, severe, and critical COVID-19 cases. Continuous variables were summarized by the mean, standard deviation (SD), and median, and group comparisons were performed with a *t*-test. Categorical variables were presented as frequencies and compared between groups with the chi^2^ test. Since the chi-squared test assumes sufficiently large expected cell counts, Fisher’s exact test was applied where necessary, particularly for categorical variables with low expected frequencies. Statistical significance was determined at a *p*-value of <0.05.

The statistical analyses were performed using STATA/MP 14 (StataCorp LLC, College Station, TX, USA) and Microsoft SQL Server 2014 (Microsoft, Redmond, WA, USA).

### 2.5. Regulatory Aspects

The study was conducted in accordance with the Declaration of Helsinki and was approved by the Ethics Committee at the University Medical Center Rostock (A2024-0100). Since the study used anonymized retrospective data from a statutory health insurance database, patient consent was waived. All data were handled and analyzed in compliance with Social Security data protection requirements.

## 3. Results

Based on the overall population of 3,254,803 individuals, 414,648 new COVID-19 cases were identified among 371,382 unique patients in the analyzed one-year period, with some individuals experiencing more than one infection. Specifically, 339,233 patients had a single COVID-19 diagnosis, while 32,149 patients had two or more infections during the study period. The corresponding age- and gender-standardized COVID-19 case rate was 13.2% for the overall SHI population (9.7 million cases per annum). Table 1 provides an overview of the baseline characteristics of patients with non-severe (i.e., non-hospitalized) COVID-19 in comparison to those affected by severe or critical courses of the disease. The average age of patients with non-severe COVID-19 in the one-year cross-sectional study period was 46.4 years, whereas severe and critical cases occurred in those who were significantly older, with a mean age of 73.6 years and 73.1 years, respectively. In terms of gender, 56.8% of the patients with non-severe COVID-19 were female, declining to 46.7% in severe cases and further to 36.6% for critical cases. Employment status was also notably different, in line with the age distribution of case severity. Most non-severe COVID-19 patients were employed (64.8%), which affects the indirect cost burden, while the majority of severe (84.1%) and critical (87.1%) cases were pensioners or retirees.

The comorbidity indices such as the Charlson Comorbidity Index (CCI) and Elixhauser Comorbidity Index, which also correlate with age, were much higher for severe and critical cases. The average CCI for non-severe cases was 1.0 compared to 4.3 for severe cases and 4.6 for critical cases. Similarly, the Elixhauser Index was significantly higher in severe (12.4) and critical (13.4) cases than in non-severe cases (2.5) (*p* < 0.001). Pre-existing high-risk conditions, as defined by STIKO, were also more prevalent in severe and critical cases (*p* < 0.001). In total, 27.2% of critical cases could be categorized as immunocompromised compared with only 8.5% of non-severe cases (*p* < 0.001). Similarly, 90.9% of severe cases and 94.7% of critical cases had other high-risk STIKO conditions, significantly higher than the 61.9% observed in non-severe cases (*p* < 0.001).

Analogously, other health issues, such as atrial fibrillation, heart failure, coronary heart disease, depression, and anxiety disorders, were more common among severe and critical cases versus non-severe cases. Pre-existing atrial fibrillation was present in 4.1% of non-severe cases but increased to 28.6% in severe cases and 27.6% in critical cases. Likewise, heart failure and coronary heart disease were significantly more common in severe and critical cases compared with non-severe COVID-19 cases (*p* < 0.001).

**Figure 1:**
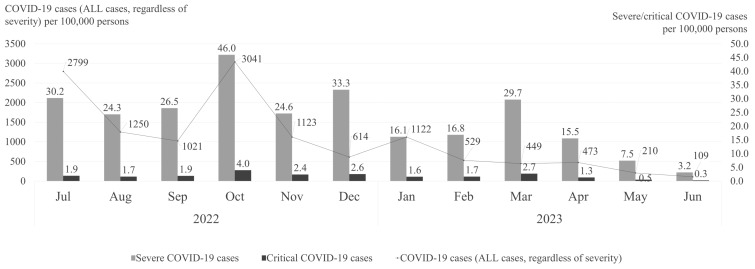
Crude rates of all COVID-19, severe COVID-19, and critical COVID-19 cases per month (July 2022–June 2023).

Figure 1 illustrates the monthly crude rates of COVID-19 cases, severe COVID-19 cases, and critical COVID-19 cases per 100,000 persons from July 2022 to June 2023. The rates of COVID-19 cases exhibited significant fluctuations over time, with notable peaks and declines. The highest incidence of COVID-19 cases occurred in October 2022.

The frequency of severe COVID-19 cases, though considerably lower than overall case rates, followed a similar seasonal trend. Peaks that clearly exceeded the mean monthly rate of 22.8 severe cases per 100,000 persons throughout the study period were observed in October 2022 (46.0 cases per 100,000 persons), December 2022 (33.3 cases per 100,000 persons), and March 2023 (29.7 cases per 100,000 persons) (Figure 1), highlighting distinct seasonal fluctuations in severe COVID-19 hospitalizations.

While consistently low throughout the study period, the critical COVID-19 case rates showed a steady presence, generally ranging between 0.3 and 4.0 cases per 100,000 persons.

**Figure 2:**
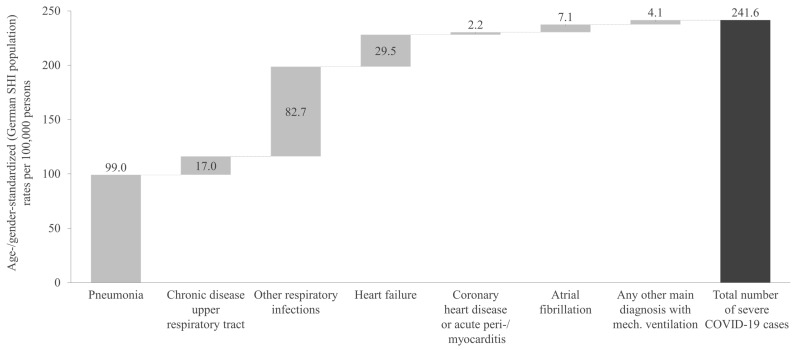
Age- and gender-standardized rate of severe COVID-19 cases during the one-year cross-sectional analysis period, categorized by main diagnosis. SHI: Statutory Health Insurance.

Figure 2 presents the age- and gender-standardized rates of severe COVID-19 cases per 100,000 persons for the overall cross-sectional study period. The total number of severe COVID-19 cases corresponds to a rate of 241.6 per 100,000 persons.

The most common severe COVID-19-related diagnosis was pneumonia, with a rate of 99.0 cases per 100,000 persons. This was followed by other respiratory infections, which showed a rate of 82.7 cases per 100,000 persons (Figure 2). Other significant diagnoses included heart failure, at 29.5 cases per 100,000 persons, and chronic upper respiratory tract diseases, at 17.0 cases per 100,000 persons. The severe/critical COVID-19 case definition referring to any other main diagnosis with mechanical ventilation showed the lowest rate, with 4.1 cases per 100,000 persons.

Of all severe COVID-19 cases, 12.4% resulted in death during hospitalization, leading to an age- and gender-standardized fatal COVID-19 rate of 28.53 per 100,000 persons. Of the severe cases with in-hospital mortality, 19.7% had a main diagnosis of pneumonia, followed by 15.0% for those with heart failure. In severe COVID-19 cases with a main diagnosis of chronic upper respiratory tract disease or other respiratory infections, a notably lower in-hospital death rate of 4.3% was observed. The age- and gender-standardized rate of critical COVID-19 cases was 20.9 per 100,000 people, corresponding to an extrapolated total of 15,365 cases in the overall SHI population during the one-year cross-sectional analysis period. For COVID-19 cases with a critical course, the in-hospital mortality was 32.2%.

**Figure 3:**
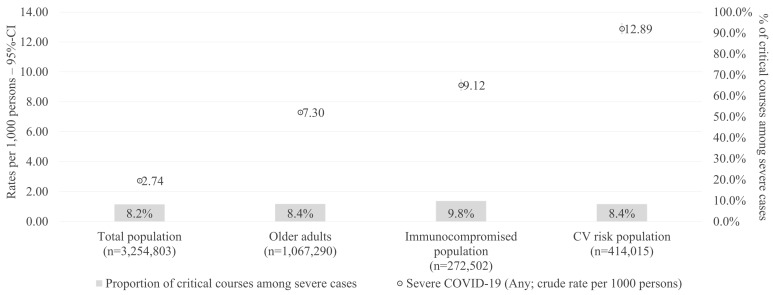
Rate of severe cases per 1000 persons (one-year cross-sectional period) in different subpopulations. CI: confidence interval; CV: cardiovascular.

In line with the observed differences in the characteristics between patients with non-severe versus severe COVID-19 cases, Figure 3 indicates that the crude rate of severe cases increased considerably in older adults, immunocompromised, and CV risk populations. Specifically, the crude rate of severe COVID-19 cases per 1000 persons was 2.74 for the total population compared with 7.30 for the older adults, 9.12 for the immunocompromised, and 12.89 for the CV risk population (Figure 3). The proportion of critical cases among severe cases was relatively similar across the different populations, ranging from 8.2% in the total population to 9.8% in the immunocompromised population.

**Figure 4:**
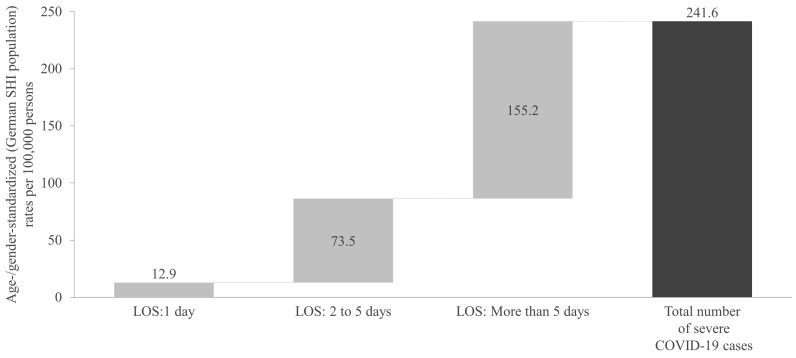
Age- and gender-standardized rate of severe COVID-19 cases during the one-year cross-sectional analysis period, categorized by hospital length of stay. SHI: Statutory Health Insurance; LOS: length of stay.

Approximately 5% of the identified severe cases had a hospital stay of only 1 day, with a rate of 12.9 per 100,000 persons (Figure 4). Approximately 30% stayed in the hospital for 2 to 5 days, with a rate of 73.5 per 100,000 persons. The remaining ~65% and thus the highest rate of severe COVID-19 cases was associated with a hospital stay of more than 5 days, at 155.2 per 100,000 persons.

As shown in Table 2, a total of 8912 COVID-19-related hospitalizations (severe or critical case definition) were observed during the one-year cross-sectional study period, amounting to 88,605 hospitalization days (an average of 9.94 inpatient days per hospitalization). The direct costs associated with these hospitalizations were EUR 64,929,586, which corresponds to 1.5% of the total hospitalization costs incurred for the observed SHI population during the one-year analysis period.

In the one-year cross-sectional study period, a total of 1,237,879 COVID-19-related outpatient GP visits were recorded, incurring associated direct costs of EUR 37,747,345. These costs accounted for 5.5% of the total outpatient GP visit expenses among the 3,254,803 insured individuals observed during the study period. Additionally, there were 407,006 COVID-19-related outpatient specialist visits, with direct costs amounting to EUR 10,983,757, representing 1.0% of the total costs for outpatient specialist visits (Table 2).

In total, 530 COVID-19-related inpatient rehabilitation cases covered by the sickness funds were observed, resulting in 10,850 rehabilitation days. The direct costs associated with these rehabilitation services were EUR 1,914,842, accounting for 1.0% of the total rehabilitation costs in the one-year analysis period (Table 2).

During the one-year cross-sectional study period, a total of 3,415,635 days absent from work due to COVID-19 were recorded in the general working population in the two German states analyzed in this study. This corresponds to an average of 1.08 days absent per person-year. The associated indirect costs of these work absences amounted to EUR 454,279,455, representing 7.5% of the total costs for all-cause work absences during the analysis period (Table 2).

To further contextualize the impact of COVID-19 on healthcare resource utilization, Supplemental Table S5 provides a detailed breakdown of COVID-19-related and all-cause costs by age group and gender for GP visits, specialist visits, and hospitalizations. COVID-19-related outpatient costs were highest among younger individuals, with GP visit costs accounting for up to 9.7% of total GP visit expenses in the 40–49 years age group. In contrast, the relative proportion of COVID-19-related hospitalization costs was highest among older adults, reaching 3.0% to 3.8% of all-cause hospitalization costs in individuals aged 80 years and older (Supplemental Table S5).

## 4. Discussion

The ROUTINE-COV19 is the first study to provide insights into the burden, outcomes, and healthcare utilization of COVID-19 within a large population covered by the SHI in Germany during the endemic respiratory disease setting. COVID-19 has become a recurring seasonal illness over a one-year period after the transition of the pandemic into an endemic phase, as seen in this study. Although the severity of the disease has decreased for most people due to widespread immunity from vaccines and prior infections, COVID-19 still poses significant risks to vulnerable populations, particularly those with pre-existing health conditions. The virus has remained a considerable burden on healthcare systems, contributing to non-severe and severe respiratory disease alongside other seasonal viruses such as influenza.

In this study of 3,254,803 individuals, clinicians diagnosed 414,648 COVID-19 cases among 371,382 unique patients over a one-year period, indicating that some individuals experienced multiple infections. The corresponding age- and gender-standardized COVID-19 case rate was 13.2% for the overall SHI population. The study highlights distinct seasonal trends, with the highest incidence of COVID-19 cases in October 2022, echoing known seasonal patterns for respiratory infections. The findings align with previously published data on COVID-19 incidence in Germany and other European countries [23,24]. For instance, data from the RKI for the same period similarly show significant fluctuations in case numbers, with peaks in autumn 2022 [24].

Severe COVID-19 cases followed a similar trend, with peak rates observed in October 2022 (46.0 cases per 100,000 persons) and March 2023 (29.7 cases per 100,000). Severe cases were primarily associated with pneumonia (41.3% of all severe COVID-19 cases) or other respiratory infections (34.0%). Notably, 12.6% of severe cases were linked to a main diagnosis of heart failure. This underscores the fact that despite being caused by a respiratory virus, COVID-19 can have remarkable implications for the cardiovascular system itself [25,26,27,28]. Although critical case rates remained consistently low (0.3 to 4.0 cases per 100,000), they persisted steadily throughout the study period. While this study focuses on observed trends using real-world claims data, mathematical modeling has also been used to examine the time-dependent dynamics of COVID-19 severity and transmission patterns. Approaches such as Markov chain modeling and other time-dependent formulations have provided additional insights into disease progression and seasonal fluctuations [29,30], complementing real-world data analysis.

Generally, the age and comorbidity profile of patients were found to be critical determinants of disease severity. The mean age of patients with severe and critical COVID-19 cases was significantly higher than those with non-severe cases, with the majority of severe and critical cases occurring among older retired individuals. This aligns with the existing literature identifying advanced age and the presence of comorbidities as strong predictors of poor outcomes in COVID-19 patients [31,32,33,34]. The significantly higher Charlson and Elixhauser Comorbidity Index scores among severe and critical cases underline the vulnerability of individuals with multiple comorbidities, particularly those with CV diseases. In our study, the rate of severe COVID-19 cases in the CV risk population (12.89 per 1000 persons) was approximately 4.7-fold higher than the rate in the total population (2.74 per 1000 persons), underscoring the high COVID-19 risk of these patients.

The breakdown of inpatient lengths of stay (1 day: 12.9 per 100,000; 2–5 days: 73.5 per 100,000; and >5 days: 155.2 per 100,000) provided further insight into the severity distribution of cases. This trend is consistent with previous reports that a significant portion of severe COVID-19 cases require extended hospitalization [32,35]. These previously analyzed cohorts also showed similar distributions in age, sex, and underlying risk factors, as seen in this study. Furthermore, our study showed that the overall in-hospital mortality among severe COVID-19 cases was 12.4%, with the highest mortality observed in patients with a primary diagnosis of pneumonia (19.7%) and heart failure (15.0%). These results underscore the significant risk of mortality associated with severe respiratory and CV complications during COVID-19 hospitalization in persons with underlying risk factors. A previous German study using nationwide inpatient samples reported an even higher in-hospital case mortality rate of 17.9% and similarly identified pneumonia diagnosis, along with older age, as a strong predictor of in-hospital death [36]. However, this study was based on 2020 data, prior to the dominance of Omicron strains and COVID-19 having become endemic. Brandt et al. recently published a German study that conducted a propensity score-matching comparison between COVID-19 patients and non-COVID-19 controls using health insurance claims data from the first quarter of 2021. The study reported that mortality more than doubled in the COVID-19 group and even tripled among patients aged 60 years and older [37]. Another German study only reviewing in-hospital mortality found case mortality rates of 8.1% (12.4% among men and 5.4% among women), which roughly aligns with the present results [38]. This previous German study, however, included data from the pandemic; this re-emphasizes the present study’s findings that endemic COVID-19 remains a continued threat to populations vulnerable to hospitalization.

Our findings reveal a substantial burden of healthcare utilization on the German healthcare system. COVID-19-related hospitalizations accounted for nearly 9000 events over the one-year period, with an average hospitalization length of 9.94 days. The direct costs associated with these hospitalizations (EUR 64.9 million) represented 1.5% of the total hospital costs within the SHI population. This reflects the significant economic impact of severe COVID-19 cases on healthcare expenditures despite the relatively small percentage of the population affected. Similarly, the claims data analysis by Brandt et al. showed that the high hospital costs of a minority of patients were the most influential driver of COVID-19-associated direct healthcare costs [37]. Although the overall percentage of patients utilizing rehabilitation services was low, the associated costs for inpatient rehabilitation further highlight the strain COVID-19 placed on post-acute care resources. The economic toll of work absences due to COVID-19 in the general working population was also concerning. With more than 3.4 million days of work lost, the indirect costs totaled EUR 454.3 million, representing 7.5% of all-cause work absenteeism costs. These data are in alignment with German work absenteeism reporting, displaying very high numbers of absences due to respiratory infections in the 2022/2023 infection season [39,40]. Work absences due to respiratory illness further increased in 2023/2024 [41], making it the leading cause of work absenteeism. This emphasizes the broader societal impact of COVID-19 beyond direct healthcare costs.

The present analysis has certain limitations that need consideration. First, the study was based on SHI claims data from Thuringia and Saxony, which may not be fully representative of the broader German population. While the dataset covers over 3 million individuals, regional variations in COVID-19 management, particularly in vaccination coverage, may influence the generalizability of our findings. As of 8 April 2023, toward the end of our study period, the national average for individuals who had received at least one booster dose was 62.6% [42]. However, according to the RKI, booster vaccination uptake in Saxony and Thuringia was considerably lower, at 50.7% and 54.3%, respectively [42]. This lower uptake may have contributed to a higher susceptibility to severe COVID-19 cases in these regions. Vaccine hesitancy and disparities in immunization coverage could have been contributing factors to the observed hospitalization rates, particularly among individuals with cardiovascular diseases and immunocompromised conditions.

Second, the retrospective design relies on the accuracy of recorded diagnostic codes and claims, which could lead to the misclassification or underreporting of certain conditions, particularly in outpatient settings. Although efforts were made to enhance data accuracy through supplementary physician surveys and case extractions from medical charts, these measures may not completely eliminate the limitations inherent in claims-based data. Additionally, the study only captures COVID-19 cases that were diagnosed by a physician, meaning that mild infections that did not require medical care and resulted in only a few days of work absenteeism are not reflected in the analysis. This might have led to an underestimation of the true incidence of COVID-19 within the study population. Similarly, for the analysis of work absences, only individuals with an official sick leave certification were considered, meaning that unreported sick leave days—such as those taken without a physician-issued note—were not captured. Given that some employment contracts in Germany allow for a limited number of uncertified sick leave days, the indirect costs associated with COVID-19-related work absences may also be underestimated. Furthermore, while this study provides valuable insights into severe COVID-19 trends in the post-pandemic phase, it is important to recognize that the available follow-up period was limited. As a result, long-term trend analyses could not be conducted. Future research with extended follow-up may help to further characterize long-term shifts in COVID-19 burden beyond seasonal variations. Furthermore, this study does not include a direct comparison of hospitalization, morbidity, or cost rates to the pre-pandemic period. While this study provides insights into the distribution of COVID-19 burden across predefined risk groups, it does not assess broader trends in hospitalizations unrelated to COVID-19. The general development of cardiovascular or respiratory hospitalizations post-COVID pandemic compared to pre-COVID times is an important research question that was not covered in this study but should be addressed in future research to better understand long-term healthcare trends. Another limitation is the lack of clinical and laboratory data, which restricted the ability to investigate specific predictors of severe COVID-19 cases. Consequently, our analysis primarily relied on diagnostic codes and comorbidity profiles, which may not capture the full spectrum of risk factors. Similarly, vaccination status was not captured in this work. Moreover, excluding individuals without continuous insurance coverage could have introduced selection bias, particularly for transient or more mobile populations. Lastly, while the study provides insights into the economic impact of COVID-19, it does not capture indirect costs beyond work absenteeism. These may include long-term productivity loss, the broader economic burden on caregivers and families, and the societal cost of lives lost due to COVID-19 [43], further underestimating the true economic impact of the disease.

## 5. Conclusions

Germany, like many other countries, has experienced fluctuating COVID-19 incidence rates as the virus has evolved. With the emergence of the Omicron variant and its subsequent subvariants, the clinical presentation of COVID-19 has shifted, leading to a predominantly milder disease course for most individuals. However, the findings of the ROUTINE-COV19 study demonstrate that even in the post-pandemic phase, COVID-19 continues to pose a significant burden, particularly for high-risk populations such as older adults and individuals with pre-existing cardiovascular conditions or immunocompromised status. Severe and critical COVID-19 cases still occurred and were associated with notably higher mortality rates, underscoring the ongoing need for targeted prevention and management strategies in these vulnerable groups.

In this study, we quantified absolute healthcare resource utilization (HCRU) and the cost burden directly related to COVID-19 diagnoses during the first year of the post-pandemic period using real-world claims data from Germany. Despite the shift to a more predictable seasonal disease phase, COVID-19-related hospitalizations, outpatient care, and rehabilitation services resulted in substantial direct costs. The economic burden was further compounded by the indirect costs of COVID-19-related work absences, which remained significant. These results indicate that even after the acute pandemic phase, COVID-19 continues to contribute meaningfully to healthcare expenditures and economic strain, particularly in high-risk groups.

## Figures and Tables

**Table 1:** Patient characteristics.

	Non-Severe COVID-19 Cases	Severe COVID-19 Cases	Critical COVID-19 Cases
N	362,786	7968	692
Age in years [mean (SD)|median]	46.4 (20.2)|46	73.6 (22.1)|81	73.1 (12.8)|75
Female gender [*n* (%)]	206,032 (56.8%)	3724 (46.7%)	253 (36.6%)
Employment status/”Type of insurance” [n (%)]			
employee	235,062 (64.8%)	374 (4.7%)	38 (5.5%)
unemployed	13,296 (3.7%)	169 (2.1%)	23 (3.3%)
pensioner/retiree	65,187 (18.0%)	6698 (84.1%)	603 (87.1%)
self-payer	14,463 (4.0%)	161 (2.0%)	15 (2.2%)
rehabilitator	576 (0.2%)	3 (0.0%)	0 (0.0%)
insured family member without an own income	34,202 (9.4%)	563 (7.1%)	13 (1.9%)
Charlson Comorbidity Index [mean (SD)|median]	1.0 (2.0)|0	4.3 (3.2)|4	4.6 (3.2)|4
Elixhauser Comorbidity Index [mean (SD)|median]	2.5 (6.6)|0	12.4 (10.9)|11	13.4 (11.1)|12
CHA_2_DS_2_-VASc score [mean (SD)|Median]	1.5 (1.6)|1	4.2 (2.0)|4	4.1 (1.9)|4
Presence of a high-risk condition—immunocompromised [n (%)]	30,816 (8.5%)	2166 (27.2%)	226 (32.7%)
Presence of a high-risk condition—others ^+^ [n (%)]	224,442 (61.9%)	7242 (90.9%)	655 (94.7%)
Pre-index AF [n (%)]	14,797 (4.1%)	2282 (28.6%)	191 (27.6%)
Pre-index HF [n (%)]	19,713 (5.4%)	2898 (36.4%)	271 (39.2%)
Pre-index CHD [n (%)]	24,332 (6.7%)	2847 (35.7%)	278 (40.2%)
Pre-index depression [n (%)]	45,876 (12.6%)	1442 (18.1%)	116 (16.8%)
Pre-index anxiety disorder [n (%)]	31,593 (8.7%)	776 (9.7%)	78 (11.3%)

All variables listed in the table were statistically significantly different (*p* < 0.05) when comparing severe and critical COVID-19 cases to non-severe cases, with the exception of “Employment: Rehabilitator” in the critical COVID-19 group. AF—atrial fibrillation; CHD—coronary heart disease; HF—heart failure; SD—standard deviation. Note: unique patients are reported in this table—in some patients, multiple COVID-19 cases occurred in the cross-sectional study period; ^+^ other than immunocompromised conditions, i.e., chronic respiratory diseases, chronic cardiovascular disease, liver disease, kidney diseases, diabetes mellitus, or other metabolic disorders, obesity, central nervous system disorders, and Trisomy 21.

**Table 2:** COVID-19-related healthcare resource utilization and associated costs during the one-year cross-sectional analysis period.

		COVID-19-Related Hospitalizations	COVID-19-Related Outpatient GP Visits	COVID-19-Related Outpatient Specialist Visits	COVID-19-Related Inpatient Rehabilitations	Days Absent from Work Due to COVID-19 (in the General Working Population)
Number of persons observed (N)		3,254,803				1,917,317
Total observational time in years		3,195,992				1,890,139
Total number of utilizations		8912	1,237,879	407,006	530	3,415,635
	Rate per person-year (95% CI)	0.003 (0.003–0.003)	0.387 (0.387–0.388)	0.127 (0.127–0.128)	0.000 (0.000–0.000)	1.807 (1.805–1.809)
Total number of inpatient days		88,605	N/A	N/A	10,850	N/A
Associated COVID-19-related costs		EUR 64,929,586.51	EUR 37,747,345.06	EUR 10,983,757.24	EUR 1,914,842.80	EUR 454,279,455.00
% of COVID-19-related costs in all-cause costs		1.5%	5.5%	1.0%	1.0%	7.5%

CI: confidence interval; GP: general practitioner; N/A: not applicable.

## Data Availability

The original contributions presented in this study are included in the article/Supplementary Materials. Further inquiries can be directed to the corresponding author.

## Supplementary Materials

The following supporting information can be downloaded at: https://www.mdpi.com/article/10.3390/v17030424/s1, Table S1: List of codes used to identify severe cases and risk populations; Table S2: Charlson Comorbidity Index; Table S3: Codes and points used to calculate the Elixhauser Comorbidity Index score; Table S4: Codes and points used to calculate CHA^2^DS^2^-VASc; Table S5: All-cause and COVID-19-related costs during the one-year cross-sectional analysis period, stratified by age and gender.

## Abbreviations

The following abbreviations are used in this manuscript:

AF	Atrial fibrillation
CCI	Charlson Comorbidity Index
CHD	Coronary heart disease
CI	Confidence interval
CNS	Central nervous system
COVID-19	Coronavirus disease 2019
CV	Cardiovascular
DRG	Diagnosis-related group
EBM	Einheitlicher Bewertungmaßstab (German uniform evaluation standard)
GP	General practitioner
HF	Heart failure
ICD-10-GM	International Classification of Disease and related health problems, 10th revision, German Modification
OPS	Operation and procedure classification system
SARS-CoV-2	Severe acute respiratory syndrome coronavirus type 2
SD	Standard deviation
SHI	Statutory Health Insurance
STIKO	Ständige Impfkommission
WHO	World Health Organization
RKI	Robert Koch Institute
LOS	Length of stay
N/A	Not applicable
