# Supplementary material for: COVID-19 Disease Burden in the Omicron Variant-Dominated Endemic Phase: Insights from the ROUTINE-COV19 Study Using Real-World German Statutory Health Insurance Data
Source: Viruses. 2025 Mar 14;17(3):424. doi: 10.3390/v17030424 (PMC11945757; doi:10.3390/v17030424)
Supplement: Supplementary file 1 [file viruses-17-00424-s001.zip › viruses-3476485-supplementary.pdf]

Supplemental Materials

Table S1: List of codes used to identify severe cases and risk populations

Sample/Subpopulation	Definition	Identification
Severe COVID-19 cases	• Hospitalizations with a main diagnosis of pneumonia (ICD-10-GM J12-J18) and a secondary COVID-19 diagnosis (ICD-10-GM U07.1!)<br>• Hospitalizations with a main diagnosis of a chronic disease of the lower respiratory tract (ICD-10-GM J40-J47) and a secondary COVID-19 diagnosis (ICD-10-GM U07.1!)<br>• Hospitalizations with a main diagnosis of respiratory infections other than those related to pneumonia or chronic disease of the lower respiratory tract (A15, A16, A48.1, J00-J06, J09-J11, J20-J22, J85, J86) and a secondary COVID-19 diagnosis (ICD-10-GM U07.1!)<br>• Hospitalizations with a main diagnosis of HF (ICD-10-GM I50) and a secondary COVID-19 diagnosis (ICD-10-GM U07.1!)<br>• Hospitalizations with a main diagnosis of CHD (ICD-10-GM I20 - I22, I24, I25) and a secondary COVID-19 diagnosis (ICD-10-GM U07.1!)<br>• Hospitalizations with a main diagnosis of acute pericarditis (ICD-10-GM I30) and a secondary COVID-19 diagnosis (ICD-10-GM U07.1!)<br>• Hospitalizations with a main diagnosis of acute myocarditis (ICD-10-GM I40) and a secondary COVID-19 diagnosis (ICD-10-GM U07.1!)<br>• Hospitalizations with a main diagnosis of AF (ICD-10-GM I48.0/1/2/9) and a secondary COVID-19 diagnosis (ICD-10-GM U07.1!)<br>• Hospitalizations with any other main diagnosis (none of the ones defined above) and a secondary COVID-19 diagnosis (ICD-10-GM U07.1!) and a documented OPS for mechanical ventilation (OPS 8-711, 8-712, 8-713, 8-714)	
**High-risk according to STIKO - Immunocompromised individuals**	Individuals who suffer from at least one of the following conditions:	
	Active treatment of solid tumor / Active neoplastic diseases	Outpatient prescription: ATC L01, L02; Inpatient treatment: OPS 8-542, 8-543, 8-544, 8-547.0, 8-547.1 (90 days pre-index) / ICD-10-GM: C00-D09
	Hematologic malignancies	Two confirmed outpatient diagnoses in two different quarters or one inpatient diagnosis of ICD-10-GM C81-C96 in the pre-index period
	Solid organ transplant	Inpatient procedure OPS 5-467.6, 5-467.9, 5-335, 5-504, 5-555, 5-375, 5-528 in the pre-index period; ICD-10-GM Z94 (transplanted organ and tissue status)
	CAR-T-cell therapy	Inpatient treatment: OPS 6-00h; Outpatient prescription: ATC L01XL03-L01XL08, L01XL90 in the pre-index period
	Blood or stem cell transplant	Inpatient procedure OPS 8-80; 5-375; 5-335; 5-504; 5-555; 5-528.2; 5-467.6; 8-860; 5-411; 8-863 in the pre-index period
	Primary immunodeficiency (e.g., common variable immunodeficiency disease, severe combined immunodeficiency, DiGeorge syndrome, Wiskott-Aldrich syndrome)	Two confirmed outpatient diagnoses in two different quarters or one inpatient diagnosis of ICD-10-GM D80-D90 in the pre-index period
	HIV infection	Two confirmed outpatient diagnoses in two different quarters or one inpatient diagnosis of ICD-10-GM B20-B24 in the pre-index period
	Active immunosuppressive therapy	Outpatient prescription: ATC L04; Inpatient treatment: OPS 8-547.3 (90 days pre-index)
**High-risk according to STIKO – other risk groups than immunocompromised individuals**	Individuals who suffer from at least one of the following conditions:	Two confirmed outpatient diagnoses in two different quarters or one inpatient diagnosis of the respective condition in the pre-index period:
	Chronic respiratory diseases	ICD-10-GM: J40-J47
	Chronic cardiovascular	ICD-10-GM: I10-I52

Sample/Subpopulation	Definition	Identification
	Liver disease	ICD-10-GM: K70-K77
	Kidney diseases	ICD-10-GM: N10-N19
	Diabetes mellitus or other metabolic disorders	ICD-10-GM: E10-E14 / E70-E90
	Obesity	ICD-10-GM: E65-E68
	CNS disorders	ICD-10-GM: G00-G99, F00-F03, F10-F19, F70-F99, I60-I69
	Trisomy 21	ICD-10-GM: Q90

AF: Atrial fibrillation; CNS: Central nervous system; CV: Cardiovascular; CHD: Coronary heart disease; EBM: Einheitlicher Bewertungsmaßstab; HF: Heart failure; HIV: Human immunodeficiency virus; ICD-10-GM: International Statistical Classification Of Diseases And Related Health Problems, 10th revision, German Modification; ICU: Intensive Care Unit; OPS: Operation and procedure classification system

Table S2: Charlson comorbidity index

No	Comorbidity	Charlson score	Definition (ICD-10-GM code)
1	Coronary artery disease	1	I20.-, I21.-, I22.-, I23.-, I24.-, I25.-
2	Congestive heart failure	1	I11.-, I50.-
3	Peripheral vascular disease	1	I73.-, I74.-, I77.-
4	Cerebrovascular disease	1	G45.-, G46.-, I6.-
5	Dementia	1	F00.-, F01.-, F02.-, F03.-, G30.-
6	Chronic pulmonary disease	1	J4x.-, J6.- w/o J67.-, J68.-, J69.-
7	Connective tissue disorder	1	M05.-, M06.-, M07.-, M08.-, M3x.-
8	Peptic ulcer disease	1	K25.-, K26.-, K27.-, K28.-
9	Mild liver disease	1	B18.-, K70.-, K73.-, K75.-
10	Diabetes mellitus without complications	1	E10.9-, E11.9-, E12.9-, E13.9.-, E14.9-
11	Hemiplegia	2	G81.-, G82.-
12	Moderate or severe renal disease	2	N17.-, N18.-, N19.-
13	Diabetes mellitus with end-organ damage	2	E10.-, E11.-, E12.-, E13.-, E14.- w/o [No .9]
14	Tumor without metastases, leukemia, lymphoma, multiple myeloma	2	Cx w/o [No 16]
15	Moderate or severe liver disease	3	K72.-, K74.-, I85.-
16	Metastatic solid tumor	6	C77.-, C78.-, C79.-, C80.-
17	AIDS	6	B20.-, B21.-, B22.-, B23.-, B24.-

Please note: The Charlson Comorbidity Index (CCI) represents the sum of all scores of the listed diagnoses that have been documented during the defined period. Each diagnosis requires at least one inpatient and/or one outpatient coding and is counted only once. The age factor (=adding 1 point to the score for each decade ≥50 years of age) is not included here!

Table S3: Codes and points used to calculate Elixhauser Comorbidity Score

Component	ICD-10 codes (Quan; König)	Points (van Walraven)
**Congestive heart failure**	I09.0, I11.0, I13.0, I13.2, I25.5, I42.0, I42.1, I42.2, I42.5, I42.6, I42.7, I42.8, I42.9, I43, I50	7
**Cardiac arrhythmias**	I44.1, I44.2, I44.3, I45.6, I47, I48, I49, R00.0, R00.1, R00.8, T82.1, Z45.00, Z45.01, Z95.0	5
**Valvular disease**	I05, I06, I07, I08, I09.1, I34, I35, I36, I37, I38, I39, Q23.0, Q23.1, Q23.2, Q23.3, Z95.2, Z95.3, Z95.4	-1
**Pulmonary circulation disorders**	I26, I27, I28.0, I28.8, I28.9	4
**Peripheral vascular disorders**	I70, I71, I73.1, I73.8, I73.9, I77.1, I79.0, I79.2, Z95.81, Z95.88, Z95.9	2
**Hypertension**	I10, I11, I12, I13, I15	0
**Paralysis**	G04.1, G11.4, G80.1, G80.2, G81, G82, G83.0, G83.1, G83.2, G83.3, G83.4, G83.9	7
**Other neurological disorders**	G10, G11, G12, G13, G20, G21, G22, G25.4, G25.5, G31.2, G31.8, G31.9, G32, G35, G36, G37, G40, G41, G93.1, G93.4, R47.0, R56	6
**Chronic pulmonary disease**	I27.8, I27.9, J40, J41, J42, J43, J44, J45, J46, J47, J60, J61, J62, J63, J64, J65, J66, J67, J68.4, J70.1, J70.3	3
**Diabetes, uncomplicated**	E10.0, E10.1, E10.9, E11.0, E11.1, E11.9, E12.0, E12.1, E12.9, E13.0, E13.1, E13.9, E14.0, E14.1, E14.9	0
**Diabetes, complicated**	E10.2, E10.3, E10.4, E10.5, E10.6, E10.7, E10.8, E11.2, E11.3, E11.4, E11.5, E11.6, E11.7, E11.8, E12.2, E12.3, E12.4, E12.5, E12.6, E12.7, E12.8, E13.2, E13.3, E13.4, E13.5, E13.6, E13.7, E13.8, E14.2, E14.3, E14.4, E14.5, E14.6, E14.7, E14.8	0
**Hypothyroidism**	E00, E01, E02, E03, E89.0	0
**Chronic renal failure**	I12.0, I31.1, N18, N19, N25.0, Z49.0, Z49.1, Z49.2, Z94.0, Z99.2	5
**Liver disease**	B18, I85, I86.4, I98.2, K70, K71.1, K71.3, K71.4, K71.5, K71.7, K72, K73, K74, K76.0, K76.2, K76.9, Z94.4	11
**Peptic ulcer disease, excluding bleeding**	K25.7, K25.9, K26.7, K26.9, K27.7, K27.9, K28.7, K28.9	0
**AIDS/HIV**	B20, B21, B22, B23, B24	0
**Lymphoma**	C81, C82, C83, C84, C85, C88, C96, C90.0, C90.2	9
**Metastatic cancer**	C77, C78, C79	12
**Solid tumor without metastasis**	C00, C01, C02, C03, C04, C05, C06, C07, C08, C09, C10, C11, C12, C13, C14, C15, C16, C17, C18, C19, C20, C21, C22, C23, C24, C25, C26, C30, C31, C32, C33, C34, C37, C38, C39, C40, C41, C43, C45, C46, C47, C48, C49, C50, C51, C52, C53, C54, C55, C56, C57, C58, C60, C61, C62, C63, C64, C65, C66, C67, C68, C69, C70, C71, C72, C73, C74, C75, C76, C80, C97	4
**Rheumatoid arthritis/collagen vascular diseases**	L94.0, L94.1, L94.3, M05, M06, M08, M12.0, M12.3, M30, M31.0, M31.1, M31.2, M31.3, M32, M33, M34, M35, M45, M46.1, M46.8, M46.9	0
**Coagulopathy**	D65, D66, D67, D68, D69.1, D69.3, D69.4, D69.5, D69.6	3
**Obesity**	E66	-4
**Weight loss**	E40, E41, E42, E43, E44, E45, E46, R63.4, R64	6
**Fluid and electrolyte disorders**	E22.2, E86, E87	5
**Blood loss anemia**	D50.0	-2
**Deficiency anemia**	D50.8, D50.9, D51, D52, D53	-2
**Alcohol abuse**	F10, E52, G62.1, I42.6, K29.2, K70.0, K70.3, K70.9, T51, Z50.2, Z71.4, Z72.1	0
**Drug abuse**	F11, F12, F13, F14, F15, F16, F18, F19, Z71.5, Z72.2	-7
**Psychoses**	F20, F22, F23, F24, F25, F28, F29, F30.2, F31.2, F31.5	0
**Depression**	F20.4, F31.3-F31.5, F32, F33, F34.1, F41.2, F43.2	-3

Table S4: Codes and points used to calculate CHA²DS²-VASc

Risk factors		Points	ICD-10 GM codes
**C**	**Congestive heart failure**	1	I50
**H**	**Hypertension**	1	I10–I15
**A**	**Age** at the time of assessment ≥75 years	2	-
**D**	**Diabetes mellitus**	1	E10–E14
**S**	Previous **stroke**, TIA, or thromboembolism	2	G45.9, I63, I74
V	**Vascular disease** (previous myocardial infarction, coronary artery disease, peripheral arterial occlusive disease, atherosclerosis of the aorta)	1	I21, I22, I25.0, I25.1, I25.2, I70.0, I70.2, I73.9
**A**	**Age** at the time of assessment between 65 - 74 years	1	-
**Sc**	**Sex category** (female)	1	-
**Maximum score**		**9**	

Table S5: All-cause and COVID-19-related costs during the one-year cross-sectional analysis period, stratified by age and gender

		Female			Male			Total		
		GP visits	Specialist visits	Hospitalizations	GP visits	Specialist visits	Hospitalizations	GP visits	Specialist visits	Hospitalizations
**Age group: 17 years and younger**	COVID-19-related	1,307,661 €	125,481 €	1,226,095 €	1,353,426 €	133,008 €	1,503,394 €	2,661,087 €	258,489 €	2,729,489 €
	All-cause	58,215,331 €	49,136,239 €	157,276,981 €	61,668,887 €	46,636,385 €	181,000,047 €	119,884,217 €	95,772,625 €	338,277,029 €
	% of COVID-19-related costs relative to all-cause costs	*2.2%*	*0.3%*	*0.8%*	*2.2%*	*0.3%*	*0.8%*	*2.2%*	*0.3%*	*0.8%*
**Age group: 18-29 years**	COVID-19-related	2,396,689 €	388,434 €	85,614 €	1,980,587 €	204,328 €	246,275 €	4,377,276 €	592,762 €	331,890 €
	All-cause	28,662,781 €	61,049,078 €	117,107,973 €	25,716,613 €	21,706,456 €	61,806,131 €	54,379,395 €	82,755,534 €	178,914,104 €
	% of COVID-19-related costs relative to all-cause costs	*8.4%*	*0.6%*	*0.1%*	*7.7%*	*0.9%*	*0.4%*	*8.0%*	*0.7%*	*0.2%*
**Age group: 30-39 years**	COVID-19-related	3,841,142 €	758,273 €	155,659 €	2,865,580 €	382,202 €	189,499 €	6,706,723 €	1,140,475 €	345,158 €
	All-cause	40,941,211 €	90,039,722 €	168,027,391 €	32,987,750 €	34,590,962 €	98,175,776 €	73,928,961 €	124,630,683 €	266,203,167 €
	% of COVID-19-related costs relative to all-cause costs	*9.4%*	*0.8%*	*0.1%*	*8.7%*	*1.1%*	*0.2%*	*9.1%*	*0.9%*	*0.1%*
**Age group: 40-49 years**	COVID-19-related	3,506,474 €	858,562 €	262,160 €	2,448,375 €	482,880 €	759,361 €	5,954,849 €	1,341,443 €	1,021,521 €
	All-cause	36,194,113 €	75,657,065 €	117,882,571 €	30,592,918 €	37,135,945 €	121,737,155 €	66,787,032 €	112,793,010 €	239,619,726 €
	% of COVID-19-related costs relative to all-cause costs	*9.7%*	*1.1%*	*0.2%*	*8.0%*	*1.3%*	*0.6%*	*8.9%*	*1.2%*	*0.4%*
**Age group: 50-59 years**	COVID-19-related	4,608,705 €	1,276,997 €	1,307,206 €	3,028,837 €	899,884 €	1,894,944 €	7,637,542 €	2,176,882 €	3,202,150 €
	All-cause	50,449,302 €	115,430,796 €	214,865,765 €	43,724,937 €	64,866,578 €	287,973,467 €	94,174,239 €	180,297,373 €	502,839,232 €
	% of COVID-19-related costs relative to all-cause costs	*9.1%*	*1.1%*	*0.6%*	*6.9%*	*1.4%*	*0.7%*	*8.1%*	*1.2%*	*0.6%*
**Age group: 60-69 years**	COVID-19-related	3,022,780 €	1,156,479 €	3,159,354 €	2,157,530 €	1,063,272 €	7,774,046 €	5,180,310 €	2,219,751 €	10,933,400 €
	All-cause	57,304,236 €	127,224,786 €	340,734,064 €	50,421,983 €	90,057,345 €	496,302,201 €	107,726,220 €	217,282,131 €	837,036,265 €
	% of COVID-19-related costs relative to all-cause costs	*5.3%*	*0.9%*	*0.9%*	*4.3%*	*1.2%*	*1.6%*	*4.8%*	*1.0%*	*1.3%*
**Age group: 70-79 years**	COVID-19-related	1,398,299 €	810,863 €	5,790,366 €	1,026,814 €	922,786 €	10,520,716 €	2,425,113 €	1,733,649 €	16,311,083 €
	All-cause	45,298,546 €	97,562,529 €	374,366,778 €	35,379,894 €	78,506,984 €	454,139,133 €	80,678,441 €	176,069,513 €	828,505,911 €
	% of COVID-19-related costs relative to all-cause costs	*3.1%*	*0.8%*	*1.5%*	*2.9%*	*1.2%*	*2.3%*	*3.0%*	*1.0%*	*2.0%*
**Age group: 80 years and older**	COVID-19-related	1,867,751 €	828,042 €	14,628,994 €	936,695 €	692,265 €	15,425,901 €	2,804,446 €	1,520,307 €	30,054,895 €
	All-cause	55,374,862 €	99,864,343 €	592,501,378 €	29,077,771 €	64,563,521 €	408,243,693 €	84,452,633 €	164,427,864 €	1,000,745,071 €
	% of COVID-19-related costs relative to all-cause costs	*3.4%*	*0.8%*	*2.5%*	*3.2%*	*1.1%*	*3.8%*	*3.3%*	*0.9%*	*3.0%*
